# g-C_3_N_4_@TiO_2_@Fe_3_O_4_ Multifunctional Nanomaterial for Magnetic Solid-Phase Extraction and Photocatalytic Degradation-Based Removal of Trimethoprim and Isoniazid

**DOI:** 10.1021/acsomega.2c01311

**Published:** 2022-06-27

**Authors:** Gokhan Sarp, Erkan Yilmaz

**Affiliations:** † Department of Analytical Chemistry, Faculty of Pharmacy, Erciyes University, 38050 Kayseri, Turkey; ‡ ERNAM-Nanotechnology Research and Application Center, Erciyes University, 38039 Kayseri, Turkey; § Technology Research & Application Center (TAUM), Erciyes University, 38039 Kayseri, Turkey; ∥ ChemicaMed Chemical Inc., Erciyes University Technology Development Zone, 38039 Kayseri, Turkey

## Abstract

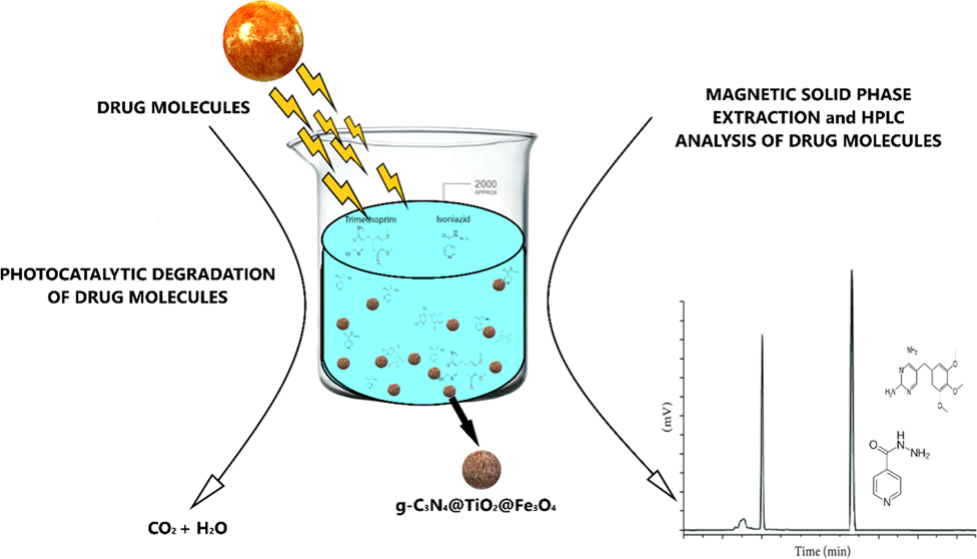

In this period when environmental pollution has become uncontrollable, the removal of drug active substances reaching the environment and the analysis of drug active substances in different matrix environments are important for both living life and a sustainable environment. Therefore, the production of multifunctional materials that can be used in these two different processes has gained importance in the literature. Based on this thought, in this study, a g-C_3_N_4_@TiO_2_@Fe_3_O_4_ multifunctional nanohybrid material was synthesized and used for magnetic solid-phase extraction (MSPE) and photocatalytic degradation of trimethoprim and isoniazid, used together in tuberculosis treatment. All analyses were performed by high-performance liquid chromatography using a diode-array detection (HPLC-DAD) system. The synthesized material was characterized by X-ray diffraction spectroscopy (XRD), Raman spectroscopy, Fourier transform infrared (FTIR) spectroscopy, Brunauer–Emmett–Teller (BET) method, ζ-potential analysis, field-emission scanning electron microscopy (FE-SEM), and energy-dispersive X-ray spectroscopy (EDX). Important analytical parameters for the MSPE method such as the pH value of the sample solution, the volume of the sample solution, the amount of the sorbent, the type and volume of the elution solvent, and extraction time were optimized. The optimized MSPE method was then applied to different environmental waters and pharmaceutical samples. The recovery percentages for these samples were found to be between 95 and 107%. For trimethoprim and isoniazid, the limit of detections (LODs) were 0.055 and 0.145 and the limit of quantifications (LOQs) were 0.167 and 0.439 ng·mL^–1^, respectively. It was observed that ∼100% of trimethoprim and isoniazid active components were photocatalytically removed from the g-C_3_N_4_@TiO_2_@Fe_3_O_4_ nanohybrid material in ∼120 min under UV light.

## 1 Introduction

The combined use of drug active ingredients for the treatment of diseases is important in the fight against diseases. Tuberculosis (TB), caused by *Mycobacterium tuberculosis* bacteria, one of the most serious diseases of the past years, is known to kill more than 2 million people annually according to the data from the World Health Organization.^1^ The fact that the treatment of TB is very difficult and very costly has brought along the use of multiple drugs. In the treatment of TB, the combination of trimethoprim (TRM) and isoniazid (INH) drug active ingredients is used.^2^ Trimethoprim is used to treat various bacterial infections of the respiratory, urinary, and gastrointestinal tract, while isoniazid is used as a prodrug in the treatment phase.^3,4^ While the analysis of these drug active ingredients in the most frequently used drug forms is important for quality control, the control of their release to the environment after use is important for the sustainability of vitality. Therefore, it is essential in this direction to develop accurate and sensitive analytical methods for the analysis of drug active ingredients used in combination in this way.

Analysis techniques for drug active ingredients include high-performance liquid chromatography (HPLC),^3^ gas chromatography–mass spectroscopy (GC–MS),^4^ liquid chromatography–mass spectroscopy (LC–MS),^5^ and high-performance thin-layer chromatography (HPTLC).^6^ However, direct analysis is not always possible due to complications such as the lowest analyte concentration that these devices can measure, higher than the amounts found in the samples studied, and/or the matrix effects of foreign species present in the sample environment.^7,8^ Therefore, for the accurate and sensitive analysis of drug active ingredients in the instrumental detection system, a sample preparation method is required to separate drug active ingredients from the matrix environment and bring their concentration to a measurable level.^9^

MSPE known as a simple and inexpensive sample preparation method has been successfully applied for the separation and enrichment of drug components.^10,11^ The synthesis and use of new generation nanoadsorbents stand out as the most important developments that lead to different perspectives in solid-phase extraction techniques. Since the basic principle in the solid-phase extraction method is based on adsorption, the large surface areas of the produced nanomaterials maximize the adsorption capacity for analytes. At the same time, for the photocatalytic removal of the analyzed pollutants, the acquisition of photocatalytic properties during the synthesis of nanoadsorbents is an important issue in the literature.^12−14^

The fact that graphite carbon nitride (g-C_3_N_4_) has a large surface area and is a photocatalytic active material has led to its frequent use in the literature.^12−14^ Titanium dioxide (TiO_2_) is widely used in photocatalytic degradation studies due to its low bandwidth, very high surface area, and high photocatalytic efficiency.^10,15−17^ Despite the above-mentioned advantages of g-C_3_N_4_ and TiO_2_ NPs, they need to be isolated quickly and easily without agglomeration from the solution environment in solid-phase extraction and photocatalytic experiment steps. One of the effective solutions for this is the modification of nanomaterials with magnetic NPs such as Fe_3_O_4_ and α-Fe_2_O_3_. In this way, nanomaterials that gain magnetic properties can be easily isolated from the solution environment at any time by applying an external magnetic field.^17^

In the light of this information, a multifunctional nanohybrid material containing g-C_3_N_4_ NPs with high adsorption and photocatalytic properties, TiO_2_ NPs with high photocatalytic properties, and Fe_3_O_4_ NPs with high magnetism was produced. The g-C_3_N_4_@TiO_2_@Fe_3_O_4_ multifunctional nanomaterial was synthesized using a simple and green solvothermal synthesis procedure. The synthesized material was successfully used for magnetic solid-phase extraction and photocatalytic degradation of trimethoprim and isoniazid drug active ingredients.

## 2 Experimental Section

### 2.1 Materials, Reagents, and Equipment

Analytical grade reagents were used throughout the experimental study. TiO_2_ NPs had a particle size of 21 nm; FeCI_3_, CH_3_COONa, urea, and ethylene glycol were obtained from Sigma-Aldrich (St. Louis). All solutions used in HPLC-DAD analyses were of LC grade purity (Sigma-Aldrich, St. Louis). A Milli-Q system deionized water system (Millipore) was used to obtain deionized water (resistivity 18.2 MΩ·cm). 200 mg·L^–1^ stock solutions of isoniazid and trimethoprim were prepared in methanol. The analysis of trimethoprim and isoniazid were performed by the HPLC-DAD system including a C18 column (150 × 4.6 mm, 5 μm particle size, and 25 °C of column temperature). The C18 column was obtained from USEM research and development company (Kayseri, Turkey). A methanol:acetate buffer mobile phase at a flow rate of 1.2 mL·min^–1^ was used. UV detection after chromatographic separation was performed at different absorption maxima depending on the used probe analyte. A photocatalytic reactor with a 400 W power of a UV lamp (Unitermrm, Turkey) was employed for the photocatalytic degradation experiments. The structure and morphology of the synthesized g-C_3_N_4_ NPs and g-C_3_N_4_@TiO_2_@Fe_3_O_4_ NPs were investigated using a field-emission scanning electron microscope (FE-SEM, Gemini 550). The crystallographic structures of g-C_3_N_4_ NPs and g-C_3_N_4_@TiO_2_@Fe_3_O_4_ NPs were illuminated by employing a Bruker AXS D8 X-ray powder diffractometer with a simple cubic lattice and Cu Kα radiation (λ = 0.15406 nm), and the scan range (2θ) was from 5 to 90°. Raman spectra of TiO_2_ NPs, g-C_3_N_4_ NPs, and g-C_3_N_4_@TiO_2_@Fe_3_O_4_ NPs were recorded using a WITec alpha 300 M dispersive Raman spectrometer consisting of a He–Ne laser system (excitation wavelength is 532 nm). A Micromeritics Gemini VII BET analyzer was used to find the surface area, pore volume, and pore width for g-C_3_N_4_@TiO_2_@Fe_3_O_4_ NPs. For this purpose, the BET-N_2_ method was used. The ζ-potentials of g-C_3_N_4_@TiO_2_@Fe_3_O_4_ NPs were measured at different pH values using a Malvern ZEN2600 Zetasizer Nano ZS.

### 2.2 Fabrication of the g-C_3_N_4_@TiO_2_@Fe_3_O_4_ Nanohybrid Material

For the production of g-C_3_N_4_ NPs, the calcination method as a simple and fast method proven in the literature was used.^15^ First, 1.0 g of urea powder was placed in a porcelain crucible and calcined in a muffle furnace at 550 °C at a heating rate of 2.3 °C·min^–1^ in for 4 h, and then cooled at room temperature. At the end of this process, slightly yellowish g-C_3_N_4_ NPs were obtained.

For the production of g-C_3_N_4_@TiO_2_@Fe_3_O_4_ NPs, 0.5 g of g-C_3_N_4_ NPs was weighed and ultrasonicated for 20 min in 20 mL of an ethylene glycol solution. Then, 0.5 g of TiO_2_ NPs was weighed in a second beaker and then ultrasonicated in 20 mL of ethylene glycol for 20 min. Next, 0.5 g of FeCl_3_ and 2 g of CH_3_COONa were weighed in a third beaker and ultrasonicated in 15 mL of ethylene glycol until the homogenization process was completed. These three mixtures obtained were collected in a beaker. After mixing in a magnetic stirrer at 900 rpm for 5 min, it was transferred into a Teflon solvothermal synthesis vessel and subjected to a solvothermal synthesis process in a solvothermal unit at 180 °C for 16 h. After 16 h, g-C_3_N_4_@TiO_2_@Fe_3_O_4_ NPs were collected by a neodymium magnet, and washing with ethanol and deionized water was performed. It was then left to dry at 80 °C in an oven.

### 2.3 Magnetic Solid-Phase Extraction Method

**Figure 1:**
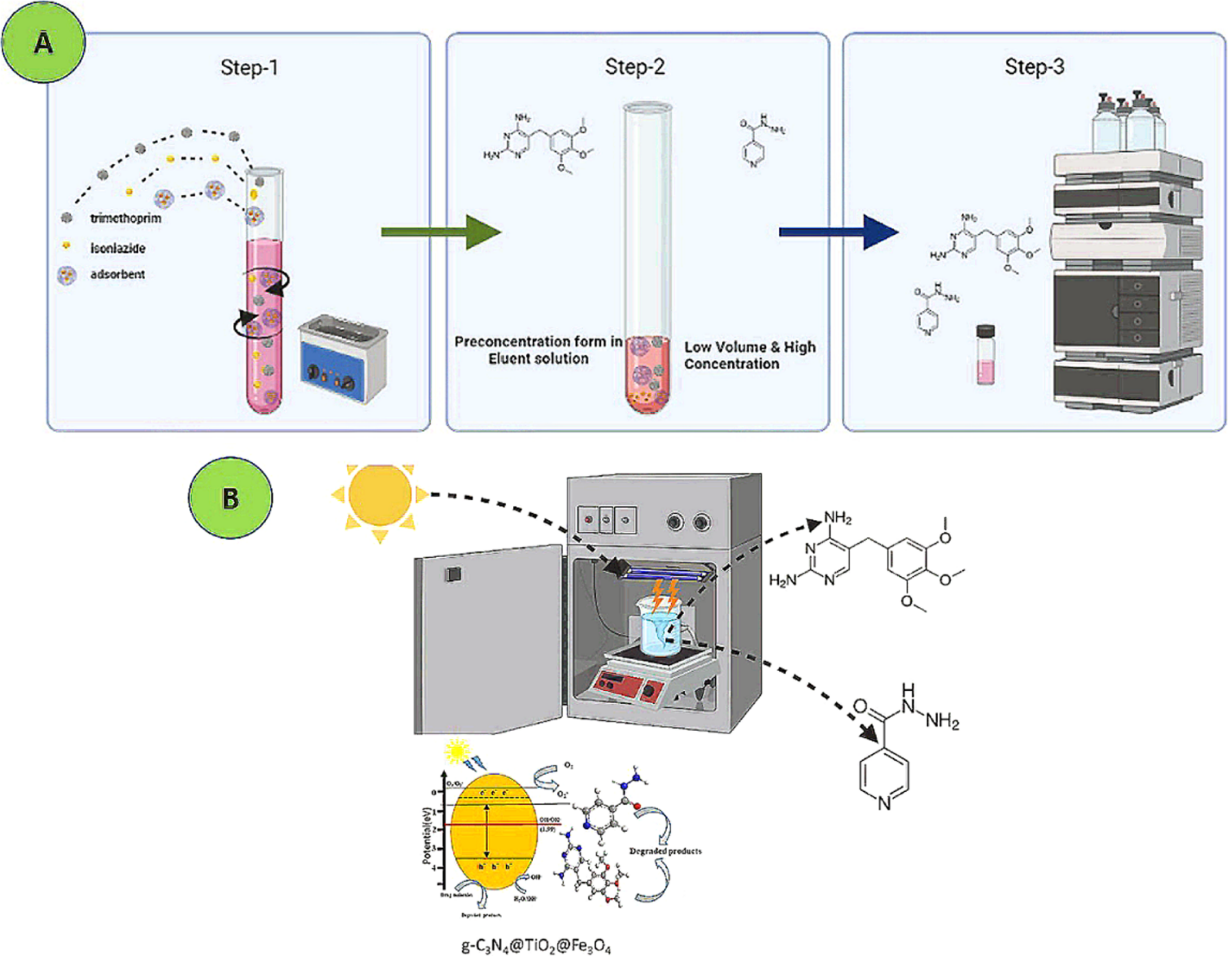
(A) Schematic representation of the MSPE/HLPC–DAD procedure and (B) photocatalytic degradation process of drug molecules.

A total of 10 mL of a model solution containing trimethoprim and isoniazid at known concentrations was prepared in a phosphate buffer medium (25 mM, pH 6.0) and 50 mg of g-C_3_N_4_@TiO_2_@Fe_3_O_4_ NPs was added to this solution. The mixture was mixed for 5 min with the help of a vortex to adsorb the drug molecules on the g-C_3_N_4_@TiO_2_@Fe_3_O_4_ NPs. Finally, g-C_3_N_4_@TiO_2_@Fe_3_O_4_ NPs were isolated from the sample solution by applying an external magnetic field with a neodymium magnet. The resulting waste phase was completely discarded. Next, g-C_3_N_4_@TiO_2_@Fe_3_O_4_ NPs were dispersed in 0.25 mL of ethanol and vortexed for 5 min to desorb drug molecules from g-C_3_N_4_@TiO_2_@Fe_3_O_4_ NPs. The same elution process was performed three times in total, and the eluents were collected in the same tube and the final eluent volume was 0.75 mL. In the last step, the eluent phase was taken using a micropipette, filtered through a 0.22 filter, and analyzed using an HPLC-DAD detection system. For the next MSPE treatment, g-C_3_N_4_@TiO_2_@Fe_3_O_4_ NPs were washed with 25 mL of water two times. The same MSPE/HPLC-DAD procedure was applied to the standard solutions and blank samples. The MSPE/HPLC-DAD procedure is schematized in Figure 1A.

### 2.4 Real Sample Applications

Real sample experiments were carried out on two different drug samples commercially available in a tablet form, lake water, seawater, and drinking water samples. Overall, 10 tablets were taken from the drugs in a tablet and homogenized in a mortar. Further, 25 mg of each sample was weighed into a flask and dissolved in 250 mL of methanol. A certain volume of these samples was taken and pure water and buffer solution were added to it, and the developed MSPE/HPLC-DAD procedure was applied. Environmental water samples were filtered through a 0.22 μm filter before use and stored at 4 °C.

### 2.5 Photocatalytic Degradation of Drug Molecules

The photocatalysis performance of g-C_3_N_4_@TiO_2_@Fe_3_O_4_ NPs was evaluated in 100 mL of a model solution medium containing known concentrations of trimethoprim or isoniazid molecules. For this purpose, 100 mg of g-C_3_N_4_@TiO_2_@Fe_3_O_4_ NPs was added to 100 mL of a model solution, and the mixture was stirred in a dark environment without light until the adsorption of the drug molecules was complete. Next, the mixture was transferred to a photocatalytic reactor and exposed to a 400 W UV halogen lamp (365 nm wavelength) located in the center of the reactor. To determine the rate of photocatalytic degradation versus time, aliquots of a 1.0 mL sample were taken for analysis at programmed time intervals (10 min/sample) and analyzed using HPLC-DAD. The degradation rate of each drug active species was calculated using the peak areas obtained from the spectra recorded by HPLC-DAD at increasing times. The photocatalytic degradation process of drug molecules is schematized in Figure 1B.

## 3 Results and Discussion

### 3.1 Characterization of the g-C_3_N_4_@TiO_2_@Fe_3_O_4_ Multifunctional Nanomaterial

**Figure 2:**
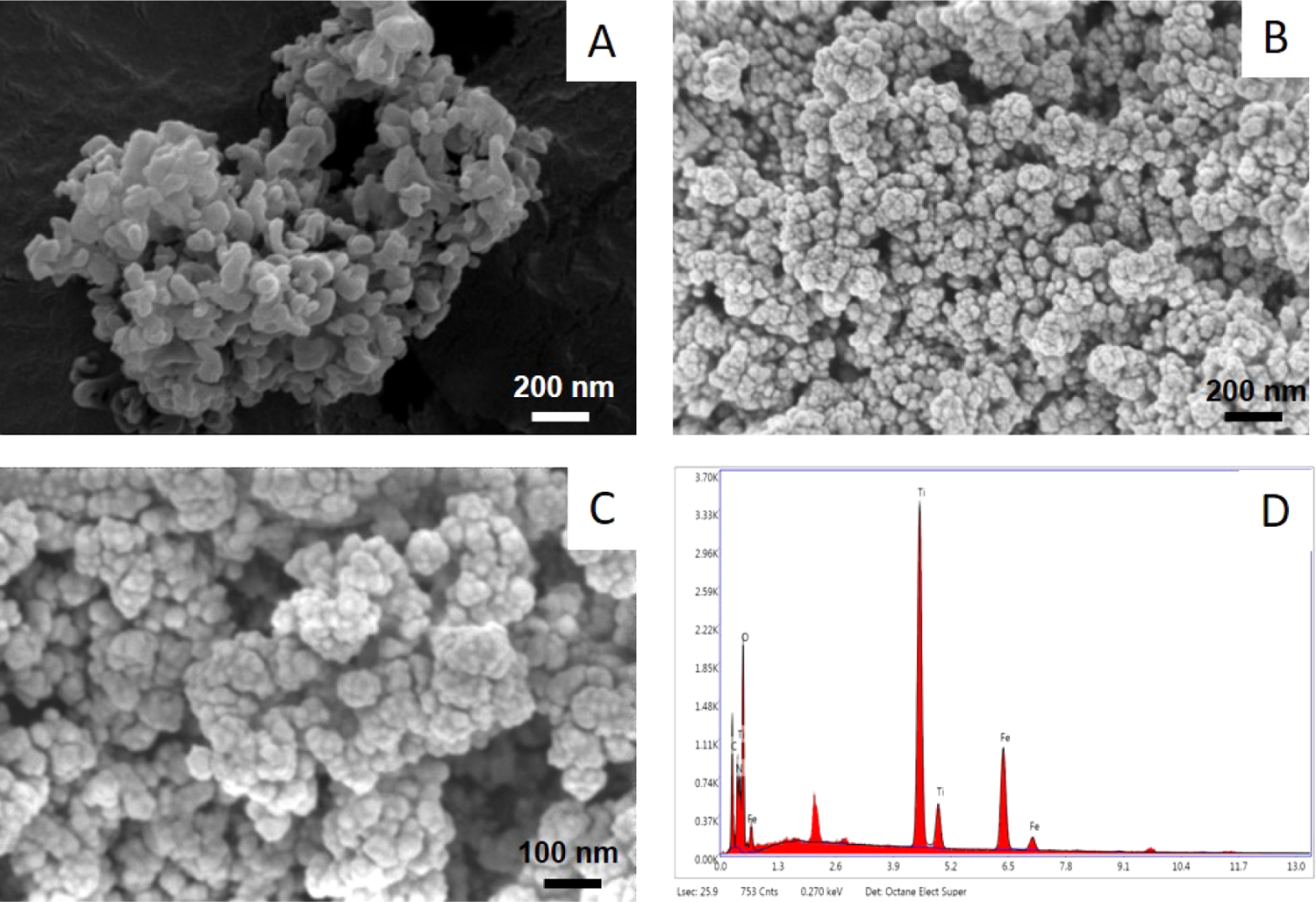
(A) SEM images of g-C_3_N_4_ and (B, C) g-C_3_N_4_@TiO_2_@Fe_3_O_4_ NPs and (D) EDX spectrum of g-C_3_N_4_@TiO_2_@Fe_3_O_4_ NPs.

FE-SEM images of the produced g-C_3_N_4_ NPs and g-C_3_N_4_@TiO_2_@Fe_3_O_4_ NPs at 25 kV at 50 000 times magnification and EDX images at 50 000–10 000 times magnification are shown in Figure 2. As shown in Figure 2, after calcination at 550 °C, the complex leaf structure of g-C_3_N_4_ NPs is seen (Figure 2A). In the images in Figure 2B,C, the final forms of the modified g-C_3_N_4_@TiO_2_@Fe_3_O_4_ NPs are seen. Fe_3_O_4_ nanospheres with sizes varying in the range of 25–120 nm were placed between the foliated structures of g-C_3_N_4_, and these nanospheres were surrounded by both g-C_3_N_4_ NPs and TiO_2_ NPs during synthesis. As can be understood from the EDX spectrum shown in Figure 2D, all of the species (C, N, O, Fe, Ti) doped in the structure can be seen. The unlabeled peak in the EDX spectrum is due to the gold plating originating from the measurement procedure in the FE-SEM. The FE-SEM and EDX results proved that the g-C_3_N_4_@TiO_2_@Fe_3_O_4_ NPs were successfully produced.

**Figure 3:**
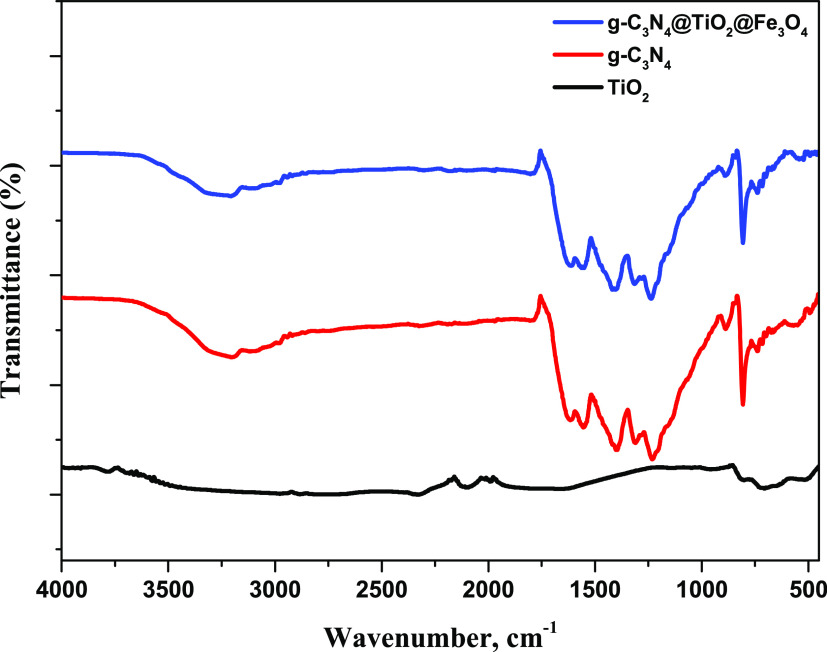
FTIR spectra of TiO_2_ NPs, g-C_3_N_4_ NPs, and g-C_3_N_4_@TiO_2_@Fe_3_O_4_ NPs.

In the FTIR spectra shown in Figure 3, a peak at 3198.4 cm^–1^ is due to N–H asymmetric tension vibrations. The several absorption peaks in the 1200–1650 cm^–1^ region correspond to typical stretching modes of C–N and C=N stretching vibrations of the CN aromatic repeating units. The two absorption peaks observed between 830 and 930 cm^–1^ are characteristic of the out-of-plane vibrations of triazine/s-triazine aromatic repeating units. The mean peak at 538.1 cm^–1^ corresponds to the Fe–O vibration. Characteristic peaks at 649 and 514 cm^–1^ are assigned to the Ti–O stretching vibrations for TiO_2_ NPs.

**Figure 4:**
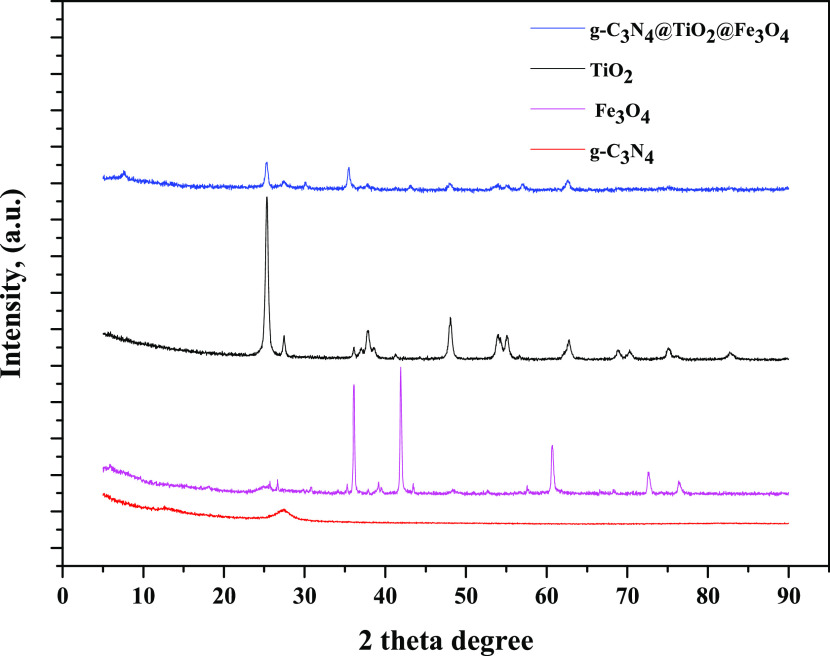
XRD patterns of Fe_3_O_4_ NPs, g-C_3_N_4,_ NPs, TiO_2_ NPs, and g-C_3_N_4_@TiO_2_@Fe_3_O_4_ NPs.

In Figure 4, XRD patterns of g-C_3_N_4_ NPs, Fe_3_O_4_ NPs, TiO_2_ NPs, and g-C_3_N_4_@TiO_2_@Fe_3_O_4_ NPs are shown. An intense peak at 2θ = 12.65 and 27.02° (JCPDS 087-1526) corresponds to graphitic planes of C_3_N_4_ NPs. The XRD peak observed at 12.65°, corresponding to the (100) plane, is due to the in-plane structural packing motif of the aromatic segments. The strongest peak at 27.02°, corresponding to the (002) plane, is due to the stacking of the conjugated aromatic system, which characterizes the interlayer *d*-spacing of g-C_3_N_4_. Characteristic peaks for anatase TiO_2_ NPs were confirmed by other studies in the literature.^16^

For the XRD spectrum of Fe_3_O_4_ NPs, 2θ = 31,0, 35.3, 43.8, 52.6, and 57.8° correspond to the planes of (220), (311), (400), (422), and (511) of the magnetite Fe_3_O_4_ NP phase (JCPDS Card No. 01-075-0449). The peaks obtained for Fe_3_O_4_ NPs are in agreement with the cubic structure of magnetite Fe_3_O_4_-NPs (space group: *Fd*-3*m*). When the spectrum of g-C_3_N_4_@TiO_2_@Fe_3_O_4_ NPs is examined, it is seen that the characteristic peaks of Fe_3_O_4_ NPs, TiO_2_ NPs, and g-C_3_N_4_ NPs are obtained by decreasing their peak intensities. This is due to the composite structure of g-C_3_N_4_@TiO_2_@Fe_3_O_4_ NPs NPs.

**Figure 5:**
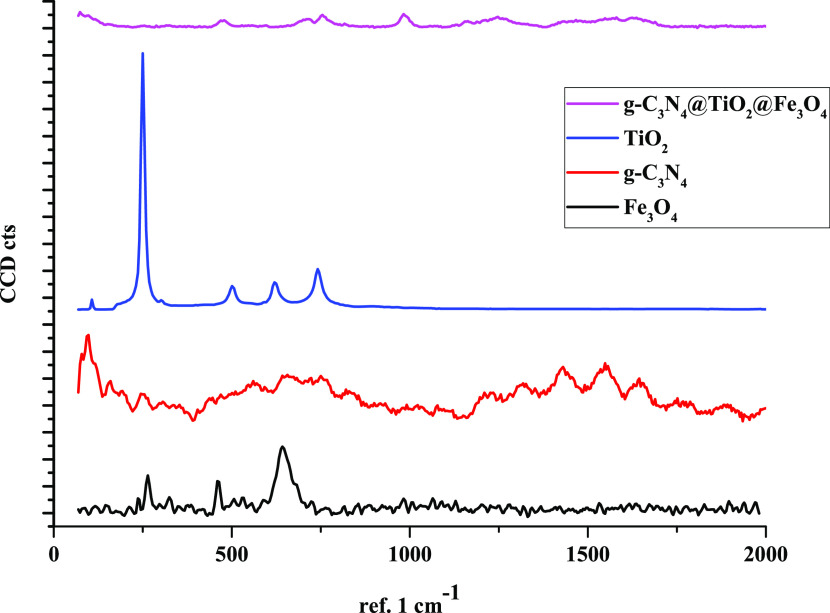
Raman spectra of TiO_2_ NPs, Fe_3_O_4_ NPs, and g-C_3_N_4_@TiO_2_@Fe_3_O_4_ NPs.

In Figure 5, the Raman spectra of pure TiO_2_ NPs, Fe_3_O_4_ NPs, and g-C_3_N_4_@TiO_2_@Fe_3_O_4_ NPs are shown. TiO_2_ NPs with an anatase form have six Raman active modes (A_1g_ + 2B_1g_ + 3E_g_) appearing at 144 cm^–1^ (E_g_), 197 cm^–1^ (E_g_), 399 cm^–1^ (B_1g_), 513 cm^–1^ (A_1g_), 519 cm^–1^ (B_1g_), and 641 cm^–1^ (E_g_).^18,19^ The Raman shift at 707 cm^–1^ is due to the respiration modes of the s-triazine ring in g-C_3_N_4_ NPs.^20^ The Raman shift at 1237 cm^–1^ is associated with the lattice vibration of the g-C_3_N_4_ crystal.

The Raman spectra of pure Fe_3_O_4_ NPs can be characterized by three peaks at 286, 482, and 664 cm^–1^. Even if the laser used in the Raman spectrometer is used at very low energy, many false peaks of hematite can be seen in the Raman spectrum of magnetite. Fe_3_O_4_ has a spinel structure, giving rise to five Raman bands: three T_2g_, one E, and one A_1g_ (Figure 5). The data obtained from the Raman spectra of the materials were confirmed by other studies in the literature.^10,16^

The BET surface area, pore volume, and pore width values for g-C_3_N_4_@TiO_2_@Fe_3_O_4_ NPs were found to be 45.5 m^2^·g^–1^, 0.29 cm^3^·g^–1^, and 254.9 Å, respectively. Materials with pore widths between 2 and 50 nm are classified as mesoporous materials. The BET isotherm results showed that mesopore characteristics of g-C_3_N_4_@TiO_2_@Fe_3_O_4_ NPs are dominant.

### 3.2 Magnetic Solid-Phase Extraction of Trimethoprim and Isoniazid

#### 3.2.1 Effect of the Sample Solution’s pH Value on MSPE Efficiency

**Figure 6:**
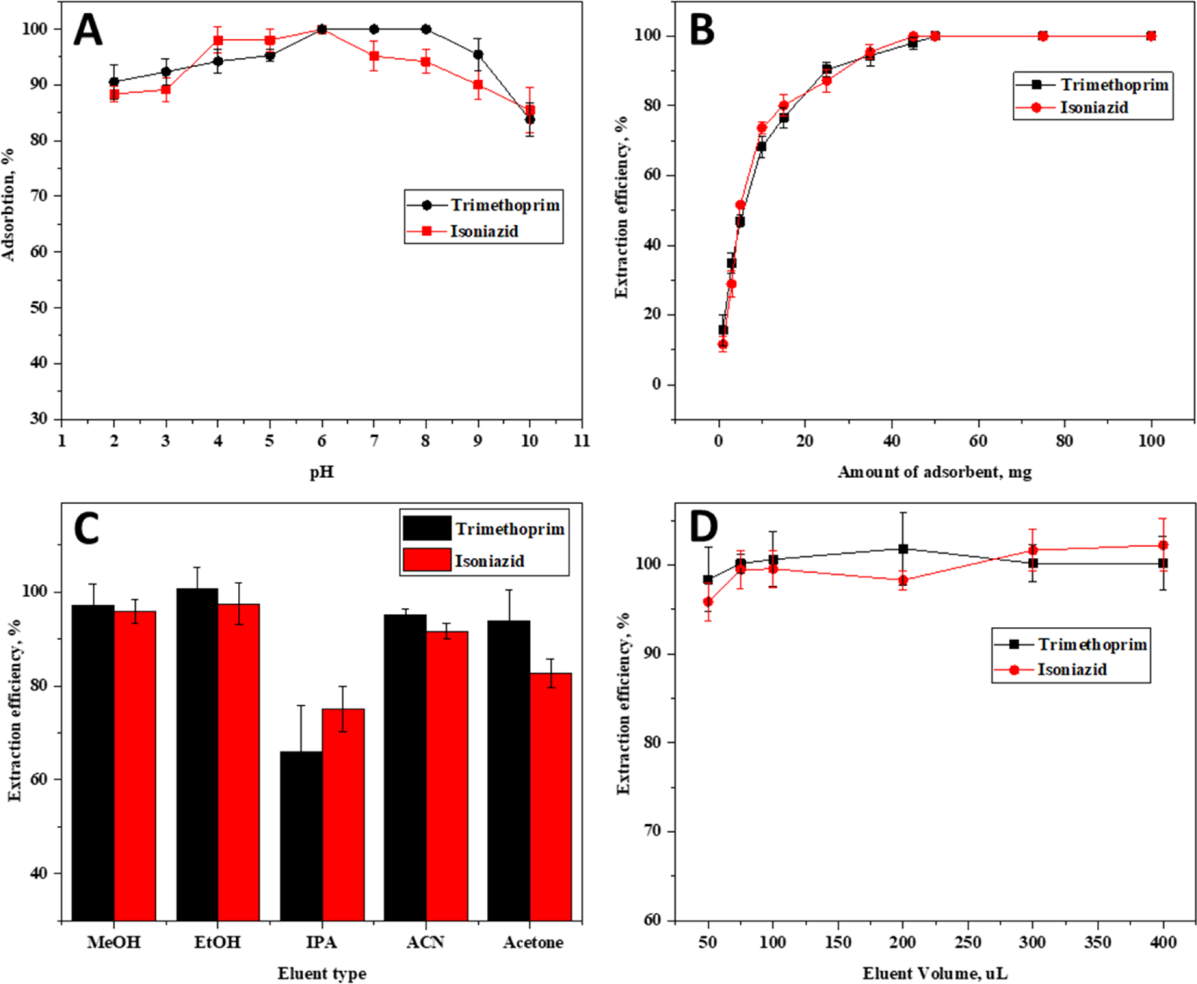
(A) Effect of sample solution pH, (B) effect of the g-C_3_N_4_@TiO_2_@Fe_3_O_4_ NP amount, and (C) effect of the eluent type and (D) eluent volume on the magnetic solid-phase extraction efficiency of trimethoprim and isoniazid (*N* = 3).

The pH value of the sample solution has a significant effect on the adsorption of the analyte molecules, as the extraction properties of the species in the sample solution medium vary depending on the pH value. Trimethoprim and isoniazid are polar compounds containing acidic and carboxylic groups, so they can be found in a neutral or ionic form. If the pH of the solution is below the p*K*_a_ value, these compounds are converted to a molecular form. The fact that most drug active species have more than one p*K*_a_ value is another important factor to be aware of. It is assumed that the adsorption efficiency increases through effective intermolecular interactions between the analyte and the adsorbent. The effect of pH of the sample solution on the adsorption efficiency of the analytes was investigated in the range of 2.0–10.0 on model solutions containing 0.25 μg·mL^–1^ of trimethoprim and isoniazid. (Figure 6A). The results obtained showed that the extraction efficiency of both active ingredients increases in parallel with the increasing pH value, reaching a maximum at pH 6.0, and the extraction efficiency decreases above pH values. Considering the results, the optimum pH value was selected as 6.0. ζ-Potential analysis was performed to determine the surface charge of the synthesized g-C_3_N_4_@TiO_2_@Fe_3_O_4_ NPs, and the results are given in Figure S1. It was observed that the surface of the g-C_3_N_4_@TiO_2_@Fe_3_O_4_ NPs was positively charged when the pH of the aqueous solution was between 2 and 4, and it was negatively charged at higher pH values. It was observed that the surface of the g-C_3_N_4_@TiO_2_@Fe_3_O_4_ NPs became more negatively charged as the pH increased. The p*K*_a_ values of trimethoprim and isoniazid are 7.12 and 1.82, respectively. While the driving force for adsorption of trimethoprim was electrostatic interactions, the driving force for adsorption of isoniazid was predicted to be interactions such as van der Waals forces and hydrogen bonding.

#### 3.2.2 Effect of the g-C_3_N_4_@TiO_2_@Fe_3_O_4_ NP Amount on MSPE Efficiency

The effect of the g-C_3_N_4_@TiO_2_@Fe_3_O_4_ NP amount on the adsorption efficiency of trimethoprim and isoniazid was investigated between 1 and 100 mg (Figure 6B). The obtained results showed that the increase in the amount of the adsorbent per unit analyte concentration caused an increase in the extraction efficiency. It was observed that the maximum extraction efficiency was reached with the addition of 50 mg of g-C_3_N_4_@TiO_2_@Fe_3_O_4_ NPs, and there was no significant change in extraction efficiency with more additions. Therefore, 50 mg of g-C_3_N_4_@TiO_2_@Fe_3_O_4_ NPs was used in subsequent experiments for both drug molecules.

#### 3.2.3 Effect of the Eluent Type and Volume on MSPE Efficiency

In solid-phase extraction methods, the elution of analytes on the adsorbent before analysis is an important step. Since trimethoprim and isoniazid are analytes in an organic form, it is appropriate to use organic solvents in their elutions. For this purpose, methanol (MeOH), ethanol (EtOH), isopropyl alcohol (IPA), acetonitrile (ACN), and acetone were used in the desorption stage of trimethoprim and isoniazid. After the elution step, the analyte concentrations in the final phase were determined by HPLC. Analyses were carried out by preparing suitable standards for each eluent phase. The quantitative recovery values for trimethoprim and isoniazid were obtained using ethanol and methanol, but the highest recoveries were reached with ethanol (Figure 6C). Therefore, ethanol was used as an eluent for subsequent experiments. It is important to perform the desorption process with the minimum possible volume of eluent to obtain a high enhancement factor, which is an important criterion for obtaining lower detection limits and consuming a low volume of an organic solvent. Desorptions of trimethoprim and isoniazid drug active ingredients adsorbed on g-C_3_N_4_@TiO_2_@Fe_3_O_4_ NPs were performed with volumes of ethanol varying from 0.5 to 4 mL and sequential extraction (Figure 6D). The results obtained showed that both active pharmaceutical ingredients were recovered quantitatively with sequential extraction using 0.25 mL of ethanol three times. The eluents were collected in the same tube and the final eluent volume was 0.75 mL.

#### 3.2.4 Effect of the Sample Solution Volume on MSPE Efficiency

The volume of the sample solution to which the method developed in sample preparation techniques can be applied is one of the most important parameters affecting the important analytical performance parameter such as the preconcentration factor, the limit of detection, and the limit of quantification. The developed MSPE method based on g-C_3_N_4_@TiO_2_@Fe_3_O_4_ was applied to sample solutions changing from 15 to 100 mL under the optimum experimental conditions. The results obtained showed that the developed MSPE method can be applied up to 75 mL of the sample volume and that the extraction yields for both drug active ingredients at higher volumes were not quantitative (Figure S2). Therefore, the developed method was applied to real samples with a maximum volume of 75 mL.

#### 3.2.5 Analytical Performance of the MSPE Method

For validation of the MSPE method, the following validation parameters were evaluated: linearity, sensitivity, precision, and preconcentration factor (Table 1). Calibration curves for trimethoprim and isoniazid were obtained after the application of the MSPE method to increasing analyte concentrations. The calculation of the LOD was based on dividing 3 times the standard deviation of the signal of the lowest concentration of the analyte concentration obtained by the slope of the calibration curve, while the LOQ was based on 10 times the standard deviation. The precision of the MSPE method was given as intraday RSD % and interday RSD % calculated by applying the MSPE method to 10 standard solutions containing 0.25 μg·mL^–1^ trimethoprim and 0.5 μg mL^–1^ isoniazid. Average recovery values were calculated by applying the MSPE method to 10 standard solutions containing 0.25 μg·mL^–1^ trimethoprim and 0.5 μg·mL^–1^ isoniazid. Analytical performance parameters for the MSPE/HPLC-DAD procedure are shown in Table 1.

**Table 1:** Analytical Performance Parameters for the MSPE/HPLC-DAD Procedure

	analytical performance results	
parameters	trimethoprim	isoniazid
calibration curve equation	*y*^a^ = 6470.9*x*^b^ + 2844.4	*y*^a^ = 4369.1*x*^b^ – 985.42
correlation coefficients (*R*^2^)	0.9994	0.9974
LOD, ng·mL^–1^	0.055	0.145
LOQ, ng·mL^–1^	0.167	0.439
intraday RSD, %	1.25	1.29
interday RSD, %	3.02	3.08
average recovery, %	98 ± 2	97 ± 4
PF	100	100

a Peak area on the HPLC-DAD system.

b Slope of the calibration curve.

#### 3.2.6 Adsorption Capacity of g-C_3_N_4_@TiO_2_@Fe_3_O_4_ NPs

Adsorption capacity is the quantitative definition of the amount of analyte adsorbed per gram amount of the adsorbent by determining the amount of the adsorbent required to quantitatively enrich the analytes in a given solution. In this study, the adsorption capacity of g-C_3_N_4_@TiO_2_@Fe_3_O_4_ NPs for trimethoprim and isoniazid was studied as another important analytical performance parameter. To find the amount of the analyte adsorbed per gram adsorbent amount, 50 mg of the adsorbent was weighed, and the MSPE method was applied at increasing concentrations of trimethoprim and isoniazid (50, 100, 250, 500, 1000, 2000, and 4000 ppm) in 10 mL of a model solution medium. After the application of the MSPE method, all samples were measured at an appropriate wavelength for each analyte in the HPLC-DAD system, and the amount of mg analyte per gram adsorbent was determined over the peak areas obtained. Adsorption capacity values for trimethoprim and isoniazid were determined as 39.9 and 47.8 mg·g^–1^, respectively.

#### 3.2.7 Accuracy of the MSPE Method

Accuracy studies for the optimized MSPE method based on g-C_3_N_4_@TiO_2_@Fe_3_O_4_ NPs were performed on lake water, seawater, drinking water, and commercially available drug preparations. For this, known amounts of analytes were added to the real samples prepared, as explained in Section 2.4, and then the developed method was applied to these samples. Addition/recovery results are shown in Table 2. The recovery percentages made in the study were found to be between 95 and 107%. The obtained recovery values showed that the developed method can be successfully applied in these matrix media.

**Table 2:** Addition/Recovery Experiments for the Analyte-Spiked Drug and Environmental Water Samples (*N* = 3)

	added, μg·mL^–1^		found, μg·mL^–1^		recovery, %	
real samples	trimethoprim	isoniazid	trimethoprim	isoniazid	trimethoprim	isoniazid
lake water		UDL		UDL^a^		
	1.5	1.5	1.49 ± 0.01^b^	1.51 ± 0.01	100 ± 1.5	101 ± 1.5
	3.0	3.0	3.10 ± 0.09	2.97 ± 0.03	103 ± 3	99 ± 3
seawater		UDL		UDL		
	2.0	2.0	1.97 ± 0.01	2.01 ± 0.01	98.5 ± 2	100.5 ± 2.0
	4.0	4.0	3.87 ± 0.06	3.79 ± 0.06	97 ± 4	95 ± 4
drink water		UDL		UDL		
	1.0	1.0	1.05 ± 0.03	1.07 ± 0.01	105 ± 1	107 ± 1
	2.0	2.0	2.01 ± 0.06	2.03 ± 0.04	101 ± 2	101.5 ± 2.0
drug-1		UDL		1.9 ± 0.03		
	2.5	2.5	2.61 ± 0.04	4.37 ± 0.02	100 ± 3	99 ± 4
	5.0	5.0	5.11 ± 0.08	6.81 ± 0.01	102 ± 5	99 ± 7
drug-2		UDL	2.21 ± 0.04	UDL		
	2.5	2.5	4.70 ± 0.09	2.44 ± 0.05	100 ± 5	98 ± 2
	5.0	5.0	7.12 ± 0.09	4.89 ± 0.10	99 ± 7	98 ± 5

a Under the detection limit.

b Mean ± standard deviation.

#### 3.2.8 Photocatalytic Degradation of Drug Molecules on g-C_3_N_4_@TiO_2_@Fe_3_O_4_ NPs

Photocatalytic degradation experiments were carried out in a sample solution medium containing drug active species. The volume of the irradiated solution was 150 mL, and the optical beam path was designed in the dark and at a distance of 10 cm from the samples in continuous magnetic stirring. A 400 W curing high-pressure UV halogen lamp was placed in the center of the reactor in a quartz sleeve. Samples in 150 mL of an aqueous phase (trimethoprim and isoniazid with 20 ppm concentration) were exposed to UV light in the presence of a photocatalyst. The time under the illumination of a UV lamp was 200 min for both species, after which 1 mL aliquots were taken for HPLC-DAD analysis at scheduled time intervals (10 min/sample). The concentration and degradation rate of the probe molecules were measured throughout the photocatalytic reaction using HPLC-DAD.

Photocatalytic efficiency was calculated using eq 1
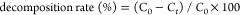
1where *C*_0_ and *C_t_* (mg·L^–1^) are the concentrations of probing molecules at the initial stage and the adsorption equilibrium concentration after irradiation at an irradiation time *t* (min), respectively. In chemical kinetics, when the reaction is first order, the reaction rate is measured based on the concentration of the reactants.

**Figure 7:**
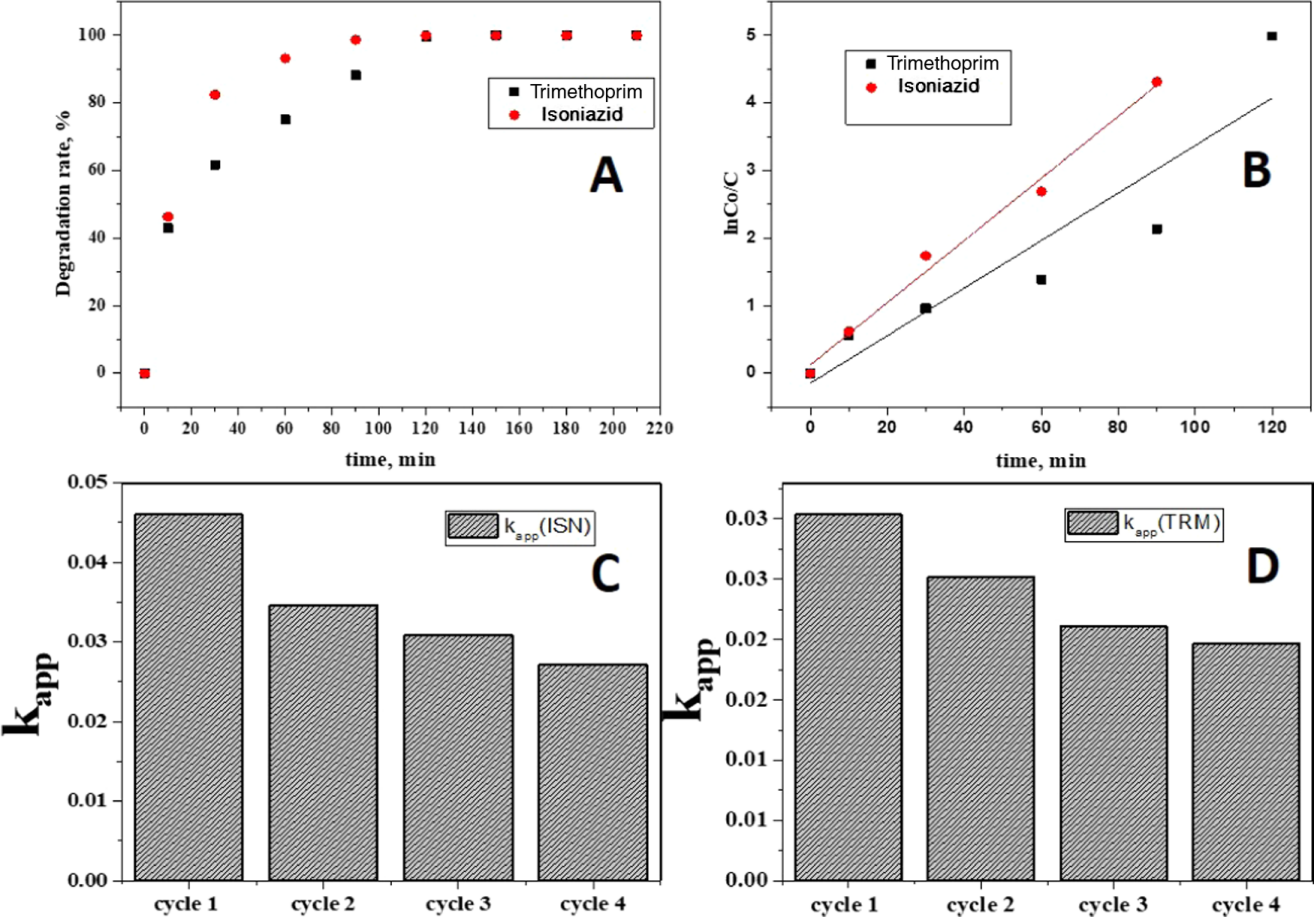
(A) Degradation rates of trimethoprim and isoniazid and (B–D) kinetics of the photocatalytic degradation rate for trimethoprim and isoniazid. The concentrations of all of the probe analytes were 20 mg·L^–1^.

So, the rate of the first-order reaction is directly proportional to the concentration of the probe molecules. The kinetics of the photocatalytic degradation rate of the probe molecules was evaluated according to the Langmuir–Hinshelwood kinetic model as given below.^8^equivalent Hinshelwood kinetic model:
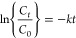
2The photocatalytic degradation efficiency of g-C_3_N_4_@TiO_2_@Fe_3_O_4_ NPs for the trimethoprim and isoniazid drug active species was analyzed according to the Langmuir–Hinshelwood kinetic model. The results obtained showed that ∼100% of trimethoprim and isoniazid were photocatalytically degraded in ∼120 and 100 min, respectively (Figure 7A). The graph of the photocatalytic removal of drug molecules versus time and the kinetic study graph are shown in Figure 7B–D. The represented fitted straight lines were plotted with a slope equal to the apparent pseudo-first-order rate constant *k* (min^–1^).

**Figure 8:**
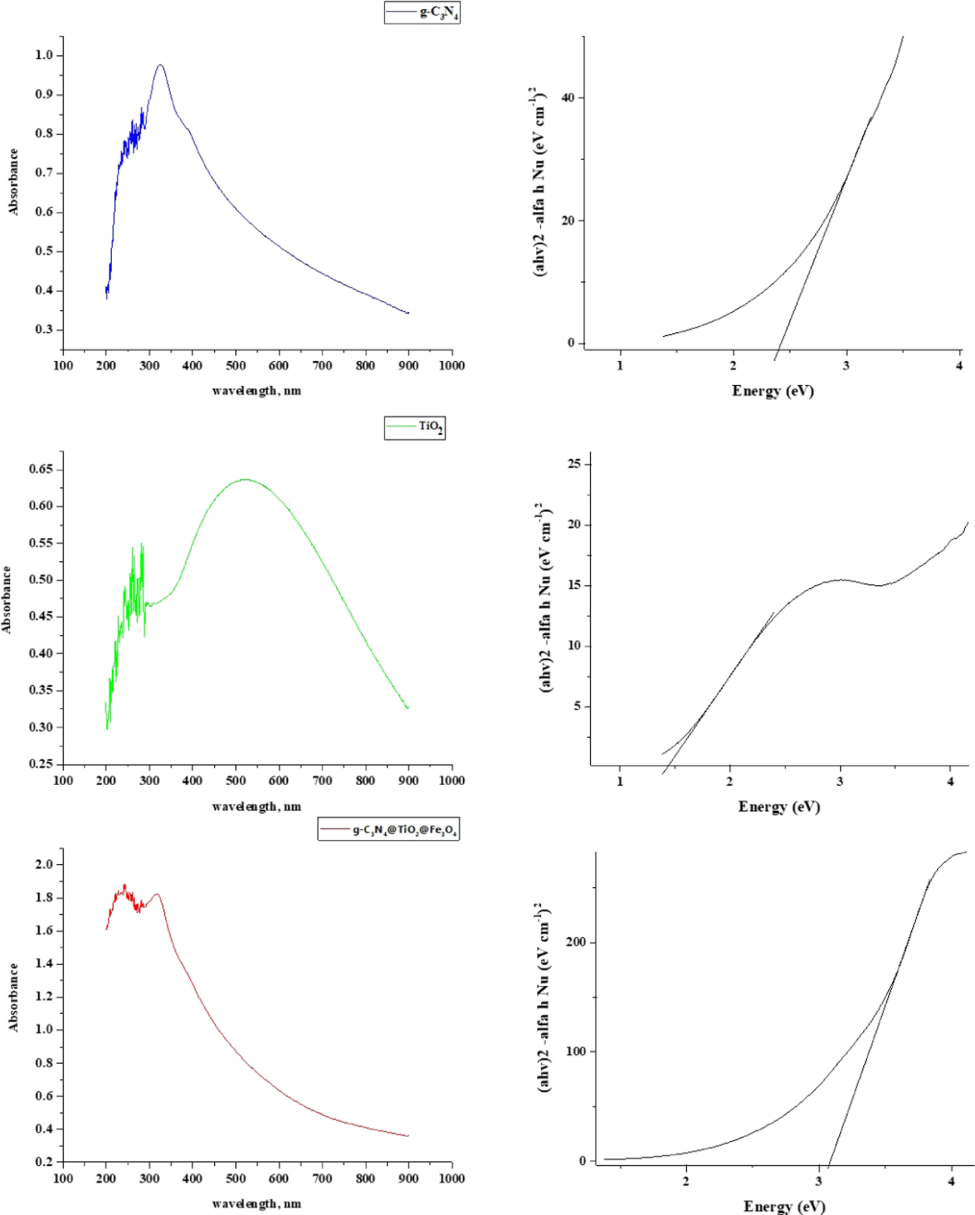
UV–vis spectra of g-C_3_N_4_ NPs, TiO_2_ NPs, and g-C_3_N_4_@TiO_2_@Fe_3_O_4_ NPs and the corresponding Tauc plot of g-C_3_N_4_ NPs, TiO_2_ NPs, and g-C_3_N_4_@TiO_2_@Fe_3_O_4_ NPs.

The UV–vis spectra were recorded for g-C_3_N_4_ NPs, TiO_2_ NPs, and g-C_3_N_4_@TiO_2_@Fe_3_O_4_ NPs (Figure 8). It was determined that TiO_2_ NPs and g-C_3_N_4_ NPs have maximum absorptions at 520 and 324.7 nm wavelengths, respectively. The absorption edges of TiO_2_ NPs and g-C_3_N_4_ NPs can be associated with the transfer of electrons from the valence band to the conduction band. The ternary nanocomposite g-C_3_N_4_@TiO_2_@Fe_3_O_4_ differed from other individual components in its absorption properties, giving a maximum absorption peak centered around 317.7 nm. This also can be attributed to the electronic transition from the valence band to the conduction band of the ternary composite and may also suggest significant interfacial contact between the g-C_3_N_4_ NPs, TiO_2_ NPs, and Fe_3_O_4_ NP components in the final nanocomposite. Also, mixing the 4s orbital of Ti with the 4s orbital of Fe may result in the formation of the conduction band at low energy. Also, Tauc’s plot used to calculate direct band gaps showed that g-C_3_N_4_ NPs, TiO_2_ NPs, and g-C_3_N_4_@TiO_2_@Fe_3_O_4_ NPs have direct band gaps of 2.39, 1.43, and 3.08 eV, respectively. These data further confirmed the formation of the intended heterojunction, enabling photocatalysis in the UV–visible light range.

**Figure 9:**
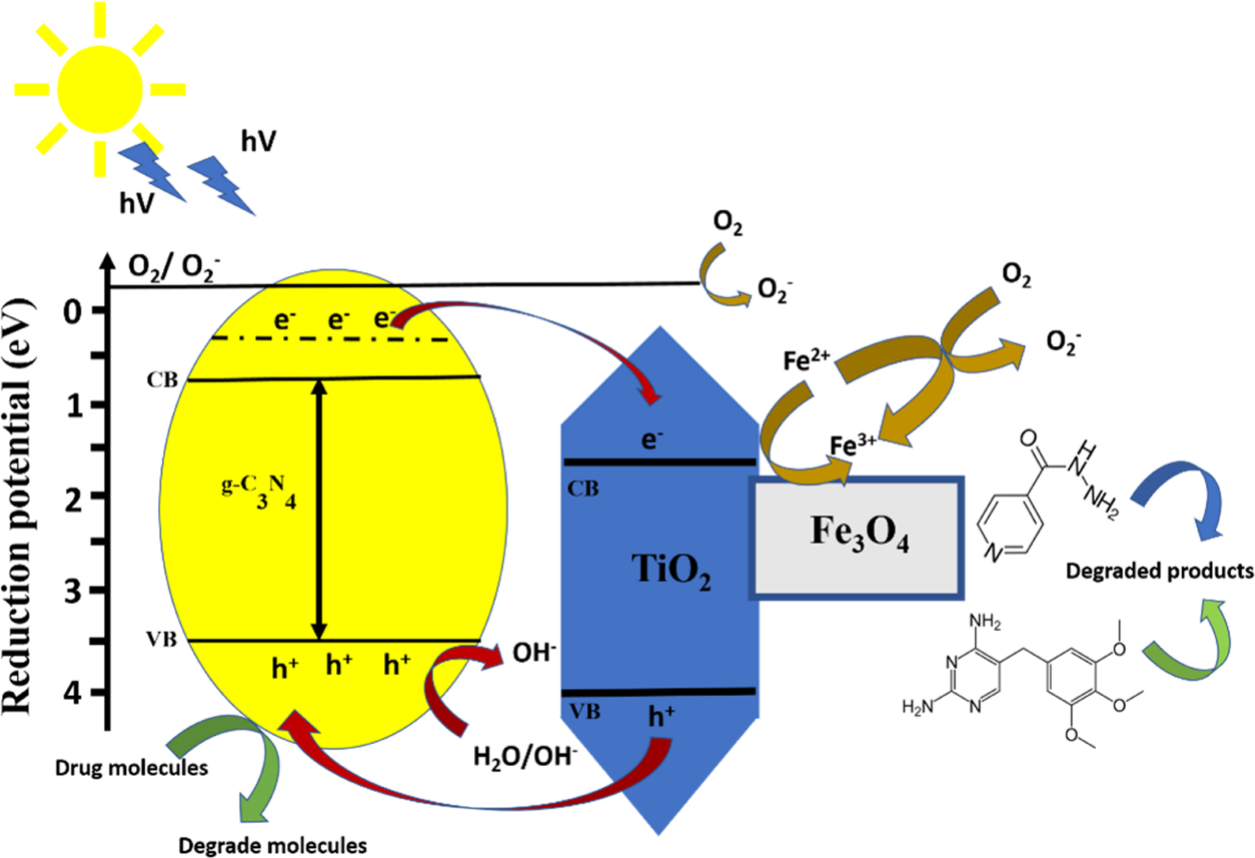
Plausible photocatalytic degradation mechanism of organic pollutants in g-C_3_N_4_@TiO_2_@Fe_3_O_4_ NPs.

The probable mechanism of the photocatalytic reaction of g-C_3_N_4_@TiO_2_@Fe_3_O_4_ NPs is shown in Figure 9. TiO_2_ NPs, from the wide-band-gap semiconductor class, have low UV light capture capacity to form light-induced electrons in the conduction band and accompanying holes in the valence band. g-C_3_N_4_ NPs in g-C_3_N_4_@TiO_2_@Fe_3_O_4_ NPs are the main component that successfully collect UV light and cause the excitation of electrons from the valence band to the conduction band. Since the conduction band of TiO_2_ has a higher reduction potential, electrons migrate from the conduction band of g-C_3_N_4_ to the conduction band of TiO_2_ due to light. In addition, however, TiO_2_ with the reduction potential of the conduction band higher than that of Fe_3_O_4_ NPs paves the way for further transfer of photogenerated electrons to the conduction band of Fe_3_O_4_ NPs. Electrons in the conduction band of TiO_2_ tend to react easily with adsorbed oxygen (O_2_) molecules to form superoxide anion radicals (^•^O_2_^–^). Also, a simultaneous hole transfer occurred from the valence band of TiO_2_ to the valence band of g-C_3_N_4_. As a result of the reaction of the holes in the valence band of g-C_3_N_4_ with water molecules (H_2_O), hydroxyl radicals (^•^OH) are formed. These radicals then react with trimethoprim and isoniazid molecules, causing their degradation. Therefore, modifying a wide-band-gap semiconductor with a narrow-band-gap semiconductor is frequently used in photocatalyst applications. The resulting hybrid photocatalyst not only absorbs the photon in the UV–visible range but also has a suppressed recombination rate of the photogenerated charges. Starting from this point, in this study, while the g-C_3_N_4_ region of the g-C_3_N_4_@TiO_2_@Fe_3_O_4_ nanohybrid material is the active site that collects light, the TiO_2_ and Fe_3_O_4_ regions play the main role in the separation of charge carriers. Thus, it led to an increase in the photocatalytic efficiency of the designed photocatalyst.

## 4 Conclusions

The fact that trimethoprim and isoniazid are used together in the treatment of diseases such as tuberculosis makes it important to analyze both of them at the same time and to remove them from environmental waters after use. In this study, which was designed with this awareness, the usability of g-C_3_N_4_@TiO_2_@Fe_3_O_4_ NPs as a magnetic adsorbent in magnetic solid-phase extraction for accurate and sensitive analysis of trimethoprim and isoniazid in different matrix media with the HPLC-DAD technique and as a magnetic photocatalyst for the degradation of trimethoprim and isoniazid in water medium was investigated. Due to its graphene-like structure, g-C_3_N_4_ NPs with high adsorption and photocatalytic properties were combined with TiO_2_ NPs with high photocatalytic properties and Fe_3_O_4_ nanoparticles with strong magnetic properties to produce a new hybrid material offering three different properties for these two different process. The obtained results proved that g-C_3_N_4_@TiO_2_@Fe_3_O_4_ NPs can be used successfully in the magnetic solid-phase extraction and the photocatalytic removal of trimethoprim and isoniazid.

## Supporting Information Available

ζ-Potential analysis figure of the synthesized g-C_3_N_4_@TiO_2_@Fe_3_O_4_ NPs and figure of the effect of the sample solution volume on MSPE efficiency (PDF)

## Notes

This manuscript is a part of Gokhan Sarp’s doctoral thesis.
