# Supplementary material for: g-C3N4@TiO2@Fe3O4 Multifunctional Nanomaterial for Magnetic Solid-Phase Extraction and Photocatalytic Degradation-Based Removal of Trimethoprim and Isoniazid
Source: ACS Omega. 2022 Jun 27;7(27):23223–33. doi: 10.1021/acsomega.2c01311 (PMC9280962; doi:10.1021/acsomega.2c01311)
Supplement: Supplementary file 1 — ao2c01311_si_001.pdf [file ao2c01311_si_001.pdf]

Supporting Information

g-C_3N_4@TiO_2@Fe_3O_4 multifunctional nanomaterial for magnetic solid phase extraction and photocatalytic degradation-based removal of trimethoprim and isoniazid

Gokhan Sarp[1,2], Erkan Yilmaz[1,2,3,4*]

[1]Department of Analytical Chemistry, Faculty of Pharmacy, Erciyes University, 38050 Kayseri, Turkey

[2]ERNAM-Nanotechnology Research and Application Center, Erciyes University, 38039 Kayseri, Turkey

[3]Technology Research & Application Center (TAUM), Erciyes University, 38039 Kayseri, Turkey

[4]ChemicaMed Chemical Inc., Erciyes University Technology Development Zone, Kayseri 38039, Turkey

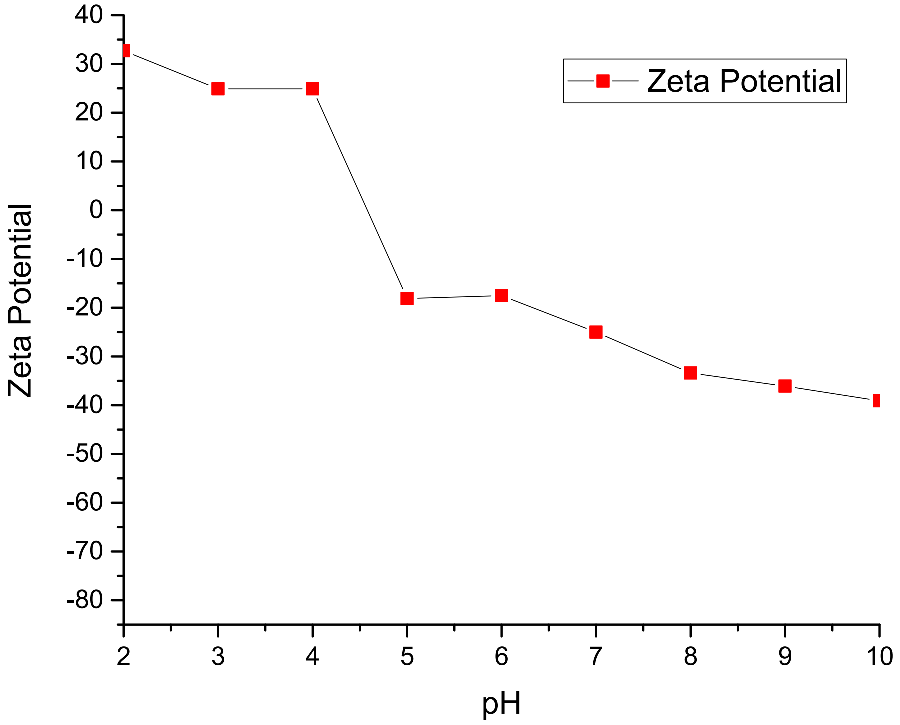

Supplementary Figure S1. Zeta potential measurement for g-C_3N_4@TiO_2@Fe_3O_4 NPs as a function of pH.

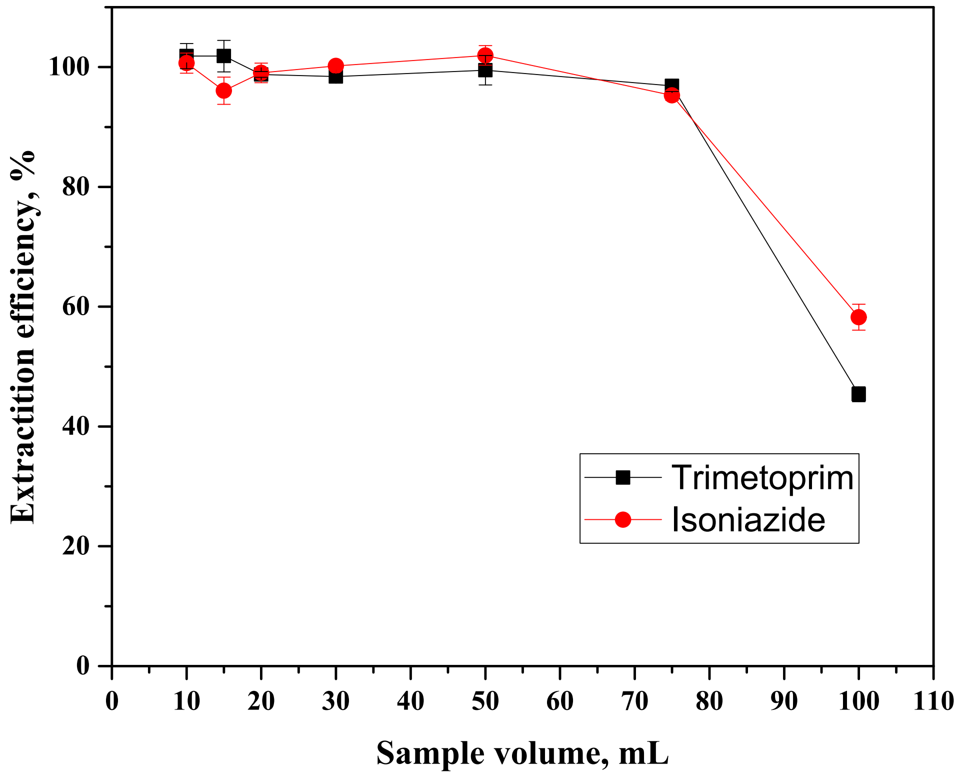

Supplementary Figure S2. Effect of sample volume on magnetic solid phase extraction efficiency of trimetoprim and isoniazid (N=3).
